# Results from one-year use of an electronic Clinical Decision Support System in a post-conflict context: An implementation research

**DOI:** 10.1371/journal.pone.0225634

**Published:** 2019-12-02

**Authors:** Andrea Bernasconi, Francois Crabbé, Ajibola Margret Adedeji, Attahiru Bello, Torsten Schmitz, Marco Landi, Rodolfo Rossi

**Affiliations:** 1 ICRC, Kyaing Tong, Myanmar; 2 HTTU, Swiss TPH, Basel, Switzerland; 3 University of Basel, Basel, Switzerland; 4 ICRC, Yola, Nigeria; 5 Adamawa State Primary Healthcare Development Agency, Adamawa, Nigeria; 6 PHC programs, ICRC, Genève, Switzerland; University of Ghana College of Health Sciences, GHANA

## Abstract

**Background:**

In 2017, the Adamawa State Primary Healthcare Development Agency introduced ALMANACH, an electronic clinical decision support system based on a modified version of IMCI. The target area was the Federal State of Adamawa (Nigeria), a region recovering after the Boko Haram insurgency. The aim of this implementation research was to assess the improvement in terms of quality care offered after one year of utilization of the tool.

**Methods:**

We carried out two cross-sectional studies in six Primary Health Care Centres to assess the improvements in comparison with the baseline carried out before the implementation. One survey was carried out inside the consultation room and was based on the direct observation of 235 consultations of children aged from 2 to 59 months old. The second survey questioned 189 caregivers outside the health facility for their opinion about the consultation carried out through using the tablet, the prescriptions and medications given.

**Results:**

In comparison with the baseline, more children were checked for danger signs (60.0% vs. 37.1% at baseline) and in addition, children were actually weighed (61.1% vs. 27.7%) during consultation. Malnutrition screening was performed in 35.1% of children (vs. 12.1%). Through ALMANACH, also performance of preventive measures was significantly improved (p<0.01): vaccination status was checked in 39.8% of cases (vs. 10.6% at baseline), and deworming and vitamin A prescription was increased to 46.5% (vs. 0.7%) and 48.3% (vs. 2.8%) respectively. Furthermore, children received a complete physical examination (58.3% vs. 45.5%, p<0.01) and correct treatment (48.4% vs. 29.5%, p<0.01). Regarding antibiotic prescription, 69.3% patients received at least one antibiotic (baseline 77.7%, p<0.05).

**Conclusions:**

Our findings highlight major improvements in terms of quality of care despite many questions still pending to be answered in relation to a full integration of the tool in the Adamawa health system.

## Introduction

In the 1990s the United Nations Children’s Fund (UNICEF) and the World Health Organization (WHO) elaborated the Integrated Management of Childhood Illness (IMCI) strategy. The aim of the IMCI guidelines development was to tackle down, at Primary Health Care (PHC) level, the five “main killers” (malaria, diarrhoea, measles, malnutrition, and pneumonia) for the population under 5 years old [1]. The strategy was adopted, integrated into the PHC system by more than 100 countries [2] and updated in 2014 [3]. The added value of the adoption of the IMCI strategy have been already widely proven and a 15% reduction in child mortality is associated to an appropriate IMCI implementation [4]. Although the benefits of the strategy are well known and understood, unfortunately in the last decades the implementation in several countries was hampered. Reasons for this were weak leadership, competition of various vertical programs, missing of a tailored tool to the epidemiological profile of the targeted population, and to the resources (staff skill level, drugs, equipment and laboratory procedures) available at PHC and a long and expensive training of the end-users [5–8]. Also, the original paper-based guidelines did not boost the implementation of IMCI for it appeared too time consuming to use in daily practice. Health workers often have the natural habit to trust rather their own ability and judgment to make diagnoses and decide treatments, quickly lost their motivation to apply to the guidelines [9–12]. The lack of routine feedback about the activities of the health workers using IMCI was also a major problem, undermining the correct implementation and adoption of the strategy, especially in the more remote areas for which IMCI was designed for [9].

Recognizing the pitfalls of the IMCI implementation and its difficulties to scaling up, an updated and digitalized version of the IMCI named ALMANACH, acronym for ALgorithms for the MANAgement of CHildhood illness, has been under development by the Swiss Tropical and Public Health Institute (Swiss TPH) since 2015, set up to use for tablets and smartphones running an android operational system [13, 14]. ALMANACH is an electronic Clinical Decision Support System (CDSS) based on a clinical decisional tree (algorithm) logic. CDSSs have shown in two meta-analysis studies to improve health workers’ performance respectively in 64% and 68% of the cases [15, 16]. Superiority of CDSSs based on IMCI (eIMCI, electronic IMCI) in comparison to the paper version (pIMCI) in terms of adherence to the protocol has also already been proven (pIMCI ranged from 61% to 98% compared to 92% to 100% under eIMCI) [11] and e-IMCI was considered easier and faster to use [17, 18]. Currently, among the existing CDSSs which are based on IMCI, eIMCI and IeDA (Integrated e-Diagnostic Approach) [19] follow entirely the IMCI. In contrast, ALMANACH and MSF eCARE, developed by Médecins sans Frontières [20], integrated also new algorithms for typhoid fever, urinary tract infection, group A streptococcal infections, and rapid diagnostic tests (RDTs) to achieve a better diagnostic precision. ePOCT, a digital solution which is currently still under development, pursues further the latter approach of integration of more innovative contents [21, 22]. ALMANACH is a derivative of eIMCI, mainly based on the version developed by Rambaud Althaus [13] who redesigned it based on evidence retrieved and results of a study on etiologies of fever in Tanzanian children seen at out-patient depts [23].

ALMANACH is used directly during the consultation of children aged from 2 to 59 months, leading the health worker from the anamnesis through the main symptoms, physical examination, in order to suggest when to refer the paediatric patient, what preventive measures are needed or required (deworming, immunization and administration of vitamin A) or treatment to offer and when to perform RDTs [14].

Objectives of ALMANACH are to guarantee a proper adherence to the protocols resulting in a better performance that can reflect a better quality of care and a rational use of the drugs (specifically antibiotics). ALMANACH is designed to work with health workers with basic medical knowledge. After three days training and minimal direct supervision, health workers are able to consult patients with the support of the tool. All clinical data collected during the consultation are sent and aggregated in the District Health Information System 2 (DHIS 2) at any time an internet connection is available. Aggregated data on DHIS2 then provide the local health care management with reliable medical information from the field, displayed on tables and graphs which are updated almost in real-time.

Several studies from Africa and Afghanistan demonstrate the advantages of adopting ALMANACH to facilitate the implementation of IMCI [14, 24–26]. The application of ALMANACH considerably improves the routine screening of children (MUAC, danger signs, deworming, vaccination schedule and vitamin A administration) and guarantees a more accurate physical examination [24]. With ALMANACH over 90% of the therapies given are in compliance with the IMCI recommendations and generally the implementation of the guidelines is facilitated [24]. ALMANACH could further be regarded as a useful tool to contrast antibiotic resistance as several studies confirmed a reduction of antibiotic prescription by 80% through its routine application [21, 24, 26].

Adamawa State in North-eastern Nigeria, approximately 3.8 million population, is one of the 36 states of the Federal Republic of Nigeria. The state is mainly inhabited by the Fulani ethnic group. Adamawa was heavily impacted by the Islamist insurgency militia Boko Haram in 2013 and a state of emergency along with neighbouring states Borno and Yobe was declared. The northern part of Adamawa state was held for six months by the insurgents with devastating consequences: many villages were destroyed and massacres carried out, leaving behind the civilian population in despair. By the end of 2014, the number of internally displaced persons in Adamawa was estimated around 450,000.

The fragile health system was, in addition, heavily affect by the armed conflict: health facilities were damaged or shut down, health staff fled, and the number of war-wounded affected noticeably on the capacity of the clinics. Until today, UNICEF is struggling to implement IMCI with the scarce health staff who remained. Furthermore, the established system of clinical training, supervision and data collection was severely disrupted. Today, Adamawa State is still troubled by frequent internal clashes between clans and farmers and breeders and the Islamic fundamentalist threat is not defeated.

In 2015, the International Committee of the Red Cross (ICRC), took the decision to support the development of ALMANACH, envisaging its implementation in conflict and post-conflict scenarios. In consideration of the situation in Adamawa, the state was chosen to gain experience with the tool by implementing ALMANACH as the standard consultation tool in PHC. ICRC’s intention met a strong political will from the Adamawa State Primary Healthcare Development Agency (ADSPHCDA) and it was decided to scale up the use of ALMANACH to state-level covering the 403 PHCC present after one-year pilot phase. Both, ICRC and ADSPHCDA, considered ALMANACH a reliable solution to improve quality of care, facilitate the training in a still volatile security context and rationalize therapies in the face of a jeopardized drug supply. ALMANACH is designed to work in remote control management and it is able to collect data when the traditional health information system stopped, for example, due to lack of staff or no capacity to access the health facilities. It guarantees a minimum of quality of care in absence of supervision and furthermore contains an on-the-job training element, through the continuous routine use of its algorithms. To back-up the knowledge of the health workers, IMCI educational material (footages, guidelines, pictures, PowerPoint presentations, and videos) have been installed on the tablets and for ease of accessibility for revision and continuous education purposes.

Under this premise, ICRC and ADSPHCDA established a partnership-project for implementation of ALMANACH, with the technical support from the Swiss TPH. Starting in December 2016, ALMANACH was introduced to 12 rural Primary Health Care Centres (PHCC) in Adamawa State, in two rounds of 6 PHCCs each. From December 2016 to February 2018, health workers consulted more than 15,000 children with the support of ALMANACH.

PHCCs in Adamawa are usually staffed with one midwife, laboratory technicians and community health extension workers (CHEW, degree after 3-year training). Rarely are community health officer workers (CHO, degree after 5-year training) and environmental health officers (EHO) in charge (EHO are in charge to implement environmental health interventions and conduct education/awareness session). The CHO is the highest education level present at the PHCC and the only staff member formally allowed to conduct thorough clinical examinations on the patient. In reality, due to chronic staff shortage also the other, less qualified, health personal at the PHCC are obliged to take care of the patients. In general, medical doctors are not found at PHC level and even the presence of a nurse is very uncommon. The PHCC’s staff is on duty 24/7.

Since the deployment of ALMANACH, programmatic data (number of consultations and diagnosis) was routinely uploaded to DHIS 2. The project manager, as well as local health authorities, analysed these data and anepidemiological bulletin is regularly published and shared with the health workers, in order to make them aware of their clinical performance. Although routine data were regularly collected, it remained unclear if and how the health workers’ practice of consulting a patient was influenced through ALMANACH: follow and adopt the guidance of the CDSS or rather use their own judgment and override recommendations they felt were not appropriate.

Our current study analyses one year of implementation of ALMANACH in Adamawa. The aim was to collect further qualitative information about the CDSS tool before its scale up to all the 403 PHCCs in the state. Thus, we consider the current experience an implementation research which is defined as investigation that “seek to understand and work in real-world or usual practice settings, paying particular attention to the audience that will use the research, the context in which implementation occurs, and the factors that influence implementation” [27].

## Materials and methods

In our study, we aimed to assess the improvement in terms of quality of care of six PHCCs one year after the introduction of ALMANACH in comparison with the results drawn from a baseline survey carried out at the same PHCCs before the implementation. In Table 1 we resume the characteristics of the six PHCCs.

**Table 1:** Characteristics of the PHCCs.

PHCC	Girei B	Lokuwa	Vinikilan	Lamurde	Muva	Betso
Catchment population	15010	12061	22359	31405	7433	3548
ALMANACH implementation	Dec 2016	Feb 2017	Dec 2016	Feb 2017	Feb 2017	Feb 2017
Average children consulted per month	200	230	205	230	100	120
**Staff**						
**Midwife**	2	2	2	0	0	0
**Nurse**	0	0	1	0	1	0
**CHEW**	2	12	4	8	4	4
**CHO**	1	3	0	3	1	1
**Lab technician**	2	1	2	1	1	1
**EHO**	1	2	0	0	0	0

During the baseline survey, 404 paediatric consultations of children aged 2–59 months were observed by a senior doctor between February and March 2017 (power 0.8, alpha 0.05) from the six PHCCs. The routine clinical activities of the health workers were scrutinized: age and sex of the patient, signs, symptoms asked or checked, physical examination performed, laboratory test requested, diagnosis and therapy given were carefully documented and compared with the IMCI protocols. The observer was instructed to interfere with the consultation only in case of serious medical malpractice to avoid introducing additional bias.

After one year, the same six PHCCs were assessed through two different surveys: one survey, the “consultation room survey” (hereafter: CRS), was undertaken inside the consultation room and it was a replica of the baseline survey carried out prior to the implementation of ALMANACH as it used the same checklist. In addition to the repetition of the baseline (CRS), we introduced a second questionnaire the “caregiver survey” (hereafter: CGS): the corresponding interview was conducted with the patients’ caregivers (parents or guardians), but without the presence of the health worker in order to minimize the observation bias present at the CRS. In the CGS interview, caregivers were interrogated if the tablet was actually used during the consultation and key information were collected about whether the relevant questions from the protocol were posed by the health worker (i.e. vaccination, deworming, vitamin A received, laboratory test requested) and about the prescription and treatment received. Through CGS it was not possible to check the quality of the physical examination, because the consultation itself was not observed. There was no link between CRS and CGS: CRS and CGS documented management of different children.

Sample size calculation was based on the consideration that the antibiotic over-prescription reduction is among the most sensitive outcomes of ALMANACH utilization. Furthermore we estimated, based on the routine data collected over 12 months, that antibiotic prescription should only be required in about 25% of the DHIS 2-recorded consultations since most of the illnesses did not have a bacterial origin. Finally, the calculation estimate resulted in a sample size of minimum 180 consultations (power 0.8, alpha 0.05) for each survey (CRS and CGS), stratified in a minimum of 30 consultations per health facility. The sample was large enough to statistically compare with precision the results globally obtained with baseline data. However, it was not suitable to compare individual results of the health facilities. CRS and CGS were carried out by a team of nurses supervised by the same health worker in charge of the baseline survey. The surveyor teams enrolled in the study all the children consulted at the PHCCs until reaching the sample (30 consultations per PHCCs) between February and March 2018.

The only criteria to be admitted into the study were a willingness to participate and an age between 2 and 59 months. Data for the CGS were collected in real-time through a tablet, using the same software (CommCare, Dimagi) used to build ALMANACH. The data digitally collected were checked daily for incongruence. Data from CRS were double entered in Microsoft Access 2016 (Microsoft Inc.). Microsoft Access allowed to have a clearly arranged overview of symptoms and treatment given to the patient: thus the patient’s clinical files were assessed one by one. At any time, data could be exported in Microsoft Excel (Microsoft Inc.) to facilitate the analysis through STATA 14 (StataCorp. 2015. Stata Statistical Software: Release 14. College Station, TX: StataCorp LP).

We have also the possibility in our study to compare the performance of the health workers using ALMANACH at the consultation (ALM+) with the health workers not using it (ALM-): before the introduction of ALMANACH, all the health workers received the same three-day refresher training about IMCI and its newly digital version introduced. Theoretical lectures about the main topics of IMCI and tablet handling were complemented by simulation training followed by a one full day coaching at PHCC level with genuine patients. At the end of the training course, each PHCC received one tablet with ALMANACH installed and all the health workers were able to manage the tool and to apply IMCI’s protocols properly. The use of ALMANACH was strongly recommended but neither mandatory nor incentivized (e.g. through salary increase). Therefore, we analysed the results from the CRS, CGS and the baseline study. Furthermore, the CRS and CGS results were stratified in two groups: one where health workers did use ALMANACH (ALM+ group) during the consultation and the other where ALMANACH was not used (ALM- group). Results were displayed in tables as proportions or medians. When required the chi-square test was used to investigate the differences, by considering a significant difference when the p-value was <0.05.

The six PHCCs shared common characteristics in terms of malaria impact, drug supply, medical and laboratory equipment. Since the implementation of ALMANACH, the health staff did not change and no major enhancements to the health facilities in terms of drug supply or equipment were realized nor changes in the workflow.

The surveys were conducted in the framework of the programmatic project assessment of ADSPHCDA and they were exempt from ethical approval. Before proceeding to any survey, verbal consent from caregivers (for CGS and CRS) and health providers (for CRS only) was obtained.

## Results

Between February and March 2018, 424 consultations of children from 2 to 59 months of age were surveyed: 235 (55.4%) for CRS and 189 (44.5%) for CGS. Most of the consultations were carried out through the use of ALMANACH (72.2%). Over a third of the children was below 12 months (35.4%) (Table 2).

**Table 2:** Profile of the sample and use of ALMANACH.

	CRS (#,%)	CGS (#,%)	TOTAL (#,%)
Children	235 (55.4)	189 (44.6)	424 (100.0)
ALM+	184 (78.3)	122 (64.5)	306 (72.2%)
***Age 2–5 month***	41 (17.4)	37 (19.6)	84 (19.8)
***6–12 month***	43 (18.3)	29 (15.3)	66 (15.6)
***13–24 month***	49 (20.9)	47 (24.9)	96 (22.6)
***25–36 month***	32 (13.6)	23 (12.2)	55 (13.0)
***37–48 month***	38 (16.2)	27 (14.3)	65 (15.3)
***49–59 month***	32 (13.6)	26 (13.7)	58 (13.7)

Most of the consultations, assessed in the CRS, were carried out by CHEWs (60.4%) followed by CHOs (16.6%) and, due to staff shortage, by other health staff for e.g. like laboratory technician and EHO (14.9%). A nurse was present in only 3.8% of the consultations.

### Medical history

At the beginning of the consultation, ALMANACH, similar to the IMCI protocol, emphasizes the importance of initial screening of the four so called “general danger signs” (DS): 1. the child is lethargic/ unconsciousness, 2. the child is convulsing or has had convulsion, 3. the child is not able to drink/breastfeed, 4. the child is vomiting everything. In case the screening for DS is positive, urgent referral of that patient shall be triggered. The results of the CRS showed that at least one DS was sought in 60% (95% IC 53.6–66.0) of the children (66.8% by considering only the children checked through ALMANACH, ALM+) and 25.9% (61) were asked for all the four DSs (Table 3). Thirty-four patients (24.1%) presented at least one DS, but most of them (73.5%) were not referred to a second level health facility. “Unable to drink” was the most asked (38.3%) and present DS. At the baseline, before the implementation of ALMANACH, 37.1% of the children were screened for the DSs, significantly lower (p < 0.01).

**Table 3:** Danger signs (at least one) sought in the children at the consultation (CRS).

	ALM+		ALM-		Tot		Baseline	
	#	%	#	%	#	%	#	%
**DS sought**	123	66.8*§	18	35.3	141	60.0§	150	37.1
**DS positive**	22	17.8	3	16.6	25	17.7	44	29.3

*Statistically significant in comparison with ALM- (p<0.01)

§ Statistically significant in comparison with baseline (p<0.01)

The patient’s history is especially important for ALMANACH to identify the children in need of preventive measures. The CDSS therefore systematically reminds health workers to ascertain if the child is missing any of the scheduled vaccinations and to check for the last vitamin A dose and deworming received. With the introduction of ALMANACH, 39.8% (95%IC 34.8–45.1) were checked for their vaccination status, 46.5% (95%IC 40.6–52.1) for receiving deworming medication (albendazole) and 48.3% for receiving vitamin A (95%IC 43.1–53.5) (Table 4). Total numbers for preventive screening were higher in the group of consultations where children were consulted with ALMANACH (ALM+) and globally they significantly improved since the baseline (p< 0.01).

**Table 4:** Preventive measures checked by the HWs stratified by the use of ALMANACH, survey and compared with the baseline.

	*CGS*				CRS		TOTAL	
		*#*	%	Unknown (#)	#	%	#	%
**Immunization**	*ALM+*	39/91	42.8*	7	82/148	55.4*	121/275	44.0*
	*ALM-*	13/59	22.0	1	3/46	6.5	16/105	15.2
	*Total*	52/150	34.6§	8	85/194	43.8§	137/344	39.8§
** **	*Baseline*						38/359	10.6
**Vitamin A**	*ALM+*	50/97	51.5*	1	104/148	70.2*	154/245	62.8*
	*ALM-*	13/59	22.0	1	2/46	4.3	15/105	14.3
	*Total*	63/156	40.4§		106/194	54.6§	169/350	48.3§
** **	*Baseline*						10/359	2.8
**Albendazole**	*ALM+*	36/78	46.2*	1	85/123	69.1*	121/201	60.2*
	*ALM-*	12/52	23.1	1	2/37	10.8	14/89	15.7
	*Total*	48/130	36.9§		89/160	55.6§	135/290	46.5§
	*Baseline*						2/273	0.7

*Statistically significant in comparison with ALM- (p<0.01)

§ Statistically significant in comparison with baseline (p<0.01)

### Physical examination

Each ALMANACH-supported consultation began with weighing of the child since ALMANACH tool calculates and adjusts therapeutic doses according to weight. With the implementation of ALMANACH 61.1% (95% IC 56.4–65.6) of the children were weighed (baseline 27.7%, p< 0.01) and more children have been weighed through the health workers using ALMANACH than among the ones not using ALMANACH (73.8% against 27.9%, p < 0.01). With patients who are 6 months or older and who weighed more than 3.5 Kg, ALMANACH asks the health worker to measure the MUAC (Children's Mid Upper Arm Circumference) for malnutrition screening. At the CRS, from 194 eligible children (6 months or older and weight more than 3.5 Kg) the MUAC was measured in 35.1% of the cases (95% IC 29.6–43.0), which is significantly higher than at the baseline (12.1%, p<0.01). Eight children (3.4%) were considered malnourished (compared to 2.5% in the baseline survey). Also, the MUAC was assessed significantly more often in the ALM+ group (43.2%) than in the ALM- group (13.0%, p<0.01).

As checking for fever is also requested by ALMANACH, at the CRS temperature was taken in 90.0% of children for whom the caregivers suspected the child to be febrile in the ALM+ group (ALM- 66.6%, p< 0.01); in the baseline study only half of the febrile children (50.1%) were measured for their temperature (p<0.01).

Through direct observation (CRS) each consultation was carefully assessed to check if the diagnosis matched the symptoms and the physical examination. More than half of the patients (58.3%, 95% IC 51.9–64.4) received a correct physical examination and a diagnosis in compliance with their clinical condition; that is an overall improvement compared to the 45.5% found at the baseline (p< 0.01).

Overlooking the respiratory rate and the hydration status were the most commonly identified gaps. In children with a history of fever and breathing problems (generally cough), IMCI suggests to count the respiratory rate to differentiate between upper respiratory tract infections (URTI) and lower respiratory tract infections (LRTI) [1, 26, 27]. In the CRS figures, out of 125 children presenting with breathing problems, respiratory rate was taken in 69 (55.2%) cases, and in 25.5% of cases at the baseline (p<0.01). Checking the hydration status is recommended for any child suffering from acute watery diarrhoea (AWD), but dehydration signs were checked in only 23 (37.1%) of the 67 children presenting with AWD (Table 5).

**Table 5:** Respiratory rate counted and hydration status checked at the consultation (CRS).

		**AWD (#)**	**Checked (#,%)**
**Hydration checked**	ALM+	55	20 (36.4)
	ALM-	7	3 (42.8)
	Total	62	23 (37.1)
	Baseline	89	42 (47.2)
		**Needed**	**Counted**
**RR Counted**	ALM+	107	65 (60.7)*
	ALM-	18	4 (22.2)
	Total	125	69 (55.2) §
	Baseline	94	24 (25.5)

*Statistically significant in comparison with ALM- (p<0.01)

§ Statistically significant in comparison with baseline (p<0.01)

### Diagnoses

Malaria, affecting almost 30% of the children, was the most prominent diagnosis, followed by URTI (25.3%) and AWD (16.9%) (Table 6). More cases of malaria were reported in the ALM- group and for most of these cases, the diagnosis was mainly based on clinical symptoms. We noticed, in comparison with the baseline, that with ALMANACH the diagnosis “other diseases” has been reduced from 20.4% to 2.8% (p< 0.01) and URTI increased from 13.9% to 24.2% (p< 0.01). In general, children consulted through ALMANACH were more likely to have multiple diagnoses than children consulted without the use of ALMANACH (1.8 vs. 1.2). In the baseline results, 127 children (23.2%) received symptoms (e.g. “fever”, “cough”, “abdominal pain”, etc.…) reported as diagnoses and to 13 children (2.4%) a treatment (even antibiotic) was prescribed without a diagnosis.

**Table 6:** Diagnoses posed at the surveys and in comparison with the baseline.

	ALM+		ALM-		Tot		Baseline	
Diagnosis	#	%	#	%	#	%	#	%
***Malaria***	140	27.0*	58	43.6§	198	30.4	166	30.3
***URTI***	144	27.7*	21	15.8	165	25.3§	76	13.9
***AWD***	94	18.1	16	12.0	110	16.9	88	16.1
***TF***	33	6.4	14	10.5	47	7.2	40	7.3
***Pneumonia***	38	7.3	4	3.0	42	6.4§	7	1.3
***Other diseases***	14	2.7§	4	3.0§	18	2.8§	112	20.4
***Skin diseases***	14	2.7	7	5.3	21	3.2	28	5.1
***measles*, *chicken pox*, *TB*, *whooping cough***	5	1.0	1	0,8	6	0.9	6	1.1
***Malnutrition***	6	1.2	2	1.5	8	1.2	10	1.8
***Dysentery***	6	1.2	0	0.0	6	0.9	1	0.2
***Anaemia***	3	0.6	1	0.8	4	0.6	0	0.0
***Group A Streptococcal Infections***	3	0.6	1	0.8	4	0.6	0	0.0
***Healthy***	3	0.6	3	2.3	6	0.9	0	0.0
***Ear infection***	3	0.6	1	0.8	4	0.6	9	1.6
***Helmentiasis***	13	2.5	0	0.0	13	2.0	5	0.9
***Total***	519	100.0	133	100.0	652	100.0	548	100.0

*Statistically significant in comparison with ALM- (p<0.01)

§ Statistically significant in comparison with baseline (p<0.01)

Based on the CRS results, we further noticed that 97.2% of typhoid fever (TF) diagnosis were in co-morbidity with malaria. It is well known that blood samples from malaria positive patients can cross-react with the Widal test generating false positive results leading to over-diagnosis of TF [28, 29]. There were also several diagnoses of TF made in children below 2 years old (14.3%), even though typhoid fever is known to be very rare in patients below 12 months and uncommon in patients below 24 months [23]. These results do not differ between the ALM+ and ALM- groups nor from the baseline, with the exception of the diagnosis among children below 24 months (44.4% vs 14.3, p< 0.01) (Table 7).

**Table 7:** Typhoid fever (TF) and Malaria (Mal) diagnosis at the CRS and at the baseline.

	CRS				Baseline			
	TF	Mal	TF+Mal	<24 months	TF	Mal	TF+Mal	<24 months
			# (%)	# (%)			# (%)	# (%)
***ALM+***	24	92	24 (100%)	4 (16.7)	N/A	N/A	N/A	N/A
***ALM-***	12	35	11 (91.6%)	1 (9.1)	N/A	N/A	N/A	N/A
**TOTAL**	36	127	35 (97.2)	5 (14.3) §	38	158	36 (94.7)	16 (44.4)

§ Statistically significant in comparison with baseline (p<0.01)

When asking the caregiver (CGS), most of them (81.5%, 95%IC 75.3–86.4) were aware of the diagnosis given to their child as it was explained to them by the health worker: in 76.0% (95%IC 54.8–68.5) of the cases, the diagnosis reported by the caregiver matched with the one recorded in the PHCC’s register.

### Laboratory findings

Through the introduction of ALMANACH, more children were tested at the laboratory than at the baseline (69.6% vs. 38.9%, p<0.01) and the health workers using ALMANACH had the tendency to refer more children to the laboratory (70.6% ALM+ vs 66.9% ALM-).

Out of 438 laboratory requests, RDT for malaria was the most requested (66.4%) followed by typhoid fever (16.2%) and packed cell volume (5%). Regarding malaria, the baseline study results reported 159 malaria cases were diagnosed but only 56% of febrile children were tested for malaria and 51 children (32.1%) were diagnosed only on the base of clinical suspicion. With the use of ALMANACH 80.9% (95%IC 75.4–85.5) of children with fever were tested (ALM- 74.7%) and diagnoses without a positive test reduced to 18.6% (ALM- 36.2%) (p<0.01) (Table 8).

**Table 8:** Appropriateness of malaria diagnosis with the use of ALMANACH and at the baseline study.

	**Surveys**				**Baseline**			
	*Febrile (#*,*%)*	*Tested for malaria (#*,*%)*	*Diagnosed (#*,*%)*	*Diagnosed w/o test (#*,*%)*	*Febrile (#*,*%)*	*Tested for malaria (#*,*%)*	*Diagnosed (#*,*%)*	*Diagnosed w/o test (#*,*%)*
***ALM+***	231 (75.5)	187 (80.9)	140 (60.6)	26 (11.2)*	N/A	N/A	N/A	N/A
***ALM-***	91 (77.1)	68 (74.7)	69 (75.8)	25 (27.5)	N/A	N/A	N/A	N/A
**TOTAL**	322 (75.9) §	255 (79.2) §	209 (64.9)	51 (15.8) §	232 (57.4)	130 (56.0)	159	51 (32.1)

*Statistically significant in comparison with ALM- (p<0.01)

§ Statistically significant in comparison with baseline (p<0.01)

Direct observation (CRS) enabled a better understanding of the (wrong) use of RDTs: 17% of the children were tested (or not tested) without adherence to guidelines, 17 children were treated for malaria without positive RDT for malaria, 37 children were tested for TF without any clear indication for this test, and only 1 out of 6 children with cough for more than 14 days was tested for TB. In the CRS sample, RDTs were more correctly used in the ALM+ group (79.5%) than in the ALM- group (62.5%) (p = <0.01).

### Treatment

The majority of the consultations (95.7%) ended with the prescription of at least one drug. Through ALMANACH 49.3% (95% IC 43.8–54.9) of the children received a prescription coherent with the diagnosis (45.8% considering ALM- group). At baseline, only the 29.5% (95% IC 25.2–34.1) of the children received the correct therapy for their diagnosis (p = <0.01) (Table 9).

**Table 9:** Therapeutic prescription coherent with the diagnosis for CRS, CGS and baseline.

	CRS		CTS		Total		Baseline	
	#	%	#	%	#	%	#	%
***ALM+***	100/184	54.3	51/122	41.8	151/306	49.3	N/A	N/A
***ALM-***	28/51	54.9	26/67	38.8	54/118	45.8	N/A	N/A
**TOTAL**	128/235	54.5§	77/189	40.7§	205/424	48.4§	119/404	29.5

§ Statistically significant in comparison with baseline

Analysis of correctness and rational prescription revealed 79.3% (95% IC 78.2–89.6) of cases were aligned with the IMCI guidelines in the ALM+ group, compared to 70.3% (95% IC 57.2–80.9) in the ALM- group (p = <0.05). At the baseline this indicator was better (85.7%, 95% IC 78.3–90.9) (Table 10).

**Table 10:** Therapeutic prescription aligned with IMCI guidelines for CRS, CGS and baseline survey.

	CRS		CGS		Total		Baseline	
	#	%	#	%	#	%	#	%
***ALM+***	79/100	79*	49/51	96.1*	128/151	84.8	N/A	N/A
***ALM-***	17/28	60.7	21/26	80.8	38/54	70.4§	N/A	N/A
**TOTAL**	96/128	75.0§	70/77	90.9	166/205	81.0	102/119	85,7

*Statistically significant in comparison with ALM-, p = <0.05)

§ Statistically significant in comparison with baseline, p = <0.05)

Regarding antibiotic prescription, 69.3% (95% IC 63.9–74.2) of children treated through ALMANACH received at least one antibiotic against 71.2% (95% IC 62.4–78.6) treated without ALMANACH. Both these figures are significantly lower in the baseline survey (77.7%, 95% IC 73.4–81.5, p <0.05) (Table 11).

**Table 11:** Patients who received at least one antibiotic at the end of the consultation.

	CRS		CGS		Total		Baseline	
	#	%	#	%	#	%	#	%
***ALM+***	126/184	68.5	86/122	70.5	212/306	69.3	N/A	N/A
***ALM-***	39/51	76.5	45/67	67.2	84/118	71.2	N/A	N/A
**TOTAL**	165/235	70.2§	131/189	69.3§	296/424	69.8§	314/404	77.7

§ Statistically significant in comparison with baseline, p = <0.05)

Out of all children who received antibiotic therapy, 65.1% (95% IC 58.5–71.2) and 71.4% (95% IC 61.0–80.0) were not in need of it in the ALM+ and ALM- group, respectively. At baseline, this figure was noticeably higher (80.3%, 95%IC 75.5–84.3, p< 0.01) (Table 12).

**Table 12:** Patients who received an antibiotic without need.

	CRS		CGS		Total		Baseline	
	#	%	#	%	#	%	#	%
***ALM+***	72/126	57.1	66/86	76.7	138/212	65.1	N/A	N/A
***ALM-***	22/39	56.4	38/45	84.4	60/84	71.4	N/A	N/A
**TOTAL**	94/165	57.0§	104/131	79.4	198/296	66.9§	252/314	80.3

§ Statistically significant in comparison with baseline, p = <0.01)

### Caregivers attitude concerning the use of the tablet

At CGS, we investigated the opinion of the caregivers regarding the use of the tablet during the consultation. When directly asked, 121 (99.2%) caregivers agreed that with the tablet the health worker investigated the health condition of the child much more carefully. Only 29 (23.8%) caregivers stated that with the tablet, the time in the waiting room increased, but surprisingly 18% claimed that with the tablet the consultation was faster.

## Discussion

With the introduction of the ALMANACH CDSS we were able to generate important results in terms of improvement of quality of care regarding preventive measures in place, physical examination and therapy prescription. In comparison with the situation at baseline, after one year, through ALMANACH more children are sought for danger signs, screened for malnutrition, checked for vaccinations, vitamin A intake and deworming; accuracy of the physical examination improved and the therapy is more coherent with the diagnosis with a slight, but significant, reduction in antibiotic prescription. Furthermore, with ALMANACH the consultation is more standardized and in compliance with the guidelines: the diagnosis “other diseases” has almost completely disappeared and the detection of URTI and LRTI had increased due to a better diagnosis accuracy as the tool remind the health worker to count for the breath rate. Also, the number of children who were diagnosed with malaria, only on the base of a clinical suspicion, has been widely reduced and generally the health workers have a more rational approach to the use of RDTs and drugs.

The implementation of ALMANACH in Adamawa State, followed a similar pathway in Afghanistan (2016–2018) but the results showed remarkable differences and thus different conclusions can be drawn. In Afghanistan, ALMANACH achieved better results: 84% of the children received a correct physical examination, more than 85% received the recommended treatment and less than 30% received antibiotic therapy [24]. In Afghanistan, ALMANACH was implemented in a more controlled environment: with merely three health facilities included and close supervision was feasible. Also, the tool was used mainly by doctors, rather than general health workers. In contrast to Afghanistan, where ALMANACH was tested for its functionality, stability, usability, and efficacy in a relatively controlled environment, in Nigeria, it was assessed for its effectiveness, reliability, interaction with the Ministry of Health system and implementation. Unfortunately, in Afghanistan, the ALMANACH program was stopped due to the critical security situation.

Whereas the Afghan study proved how much ALMANACH could support the improvement of quality of care, the results from the surveys carried out in Africa, assessing the work of health workers employed by the government and working in rural areas with little to no support, provide a picture which reflects much better the real-life working circumstances for medical care providers in these geographical regions.

Our recent findings differ also from results reported by Shao et al. [26] who found 15.4% antibiotic prescription in Tanzania. Similar to Afghanistan, in Tanzania, ALMANACH was implemented under controlled conditions where the clinical guidelines were strictly applied under a close supervision.

Despite ALMANACH stating unmistakably not giving antibiotic treatment in case of a likely viral infection, our results confirm that most HWs rather continue with their previous routine and ignore evidence-based recommendations provided by the CDSS. Reasons behind this behaviour are not fully understood and need to be further investigated. Possibly, their acting is partly supported by the ambiguous recommendation of IMCI guidelines to provide appropriate antibiotic treatment for an unidentified bacterial cause of fever to febrile children that tested negative for malaria [1]. In the presented results, out of 652 diagnoses presented, less than 20% (pneumonia, typhoid fever, dysentery, ear infection) were in need of an antibiotic treatment, all the remaining illnesses received antibiotics without evidence. Also of note, TF alone counted for 36% of diagnoses with required antibiotics. We assume TF likely to be over-diagnosed in our sample, caused by unjustified use of the RDT, resulting in poor sensitivity. In terms of reduction of antibiotic over-prescription, the presented results are in the range of achieved reduction by the IeDA in Burkina Faso that estimates a 6% to 15% reduction [19].

In our study, we tried to minimize the observational bias by introducing the CGS but a full elimination of the effect cannot be assured. Moreover, despite all HWs underwent a communal and standardized training and no changes happened at the PHCCs throughout the observed year, we cannot exclude a possible cluster effect as some health workers or PHCCs could work better than others.

Apart from the above discussed survey results, we can draw some further considerations from our one-year implementation experience in Adamawa State with ALMANACH. These lessons-learnt could be particularly important during the further implementation process with the foreseen scaling up of the intervention to the whole state. Our considerations focus on the three main groups who are benefiting from the use of ALMANACH: 1) health workers, 2) health managers, and 3) patients.

1) Regarding health workers, their commitment to use the tool should never has been underestimated and their commitment should be built as soon as possible [30, 31]. If possible, the use of the tablets should become an essential element in health workers daily working routine. A continuous use of the tablet can offer further possibilities to public health agents, beyond the advantages provided through ALMANACH: tablet use embedded in routine medical care can serve as a door-opener for the implementation of further technologies such as eLearning, drug supply management, Health Information Systems (HIS), outbreak prone diseases notification and telemedicine. We tried to encourage health workers to use the tablet by providing them with access to the aggregated medical data, underlying statistics, and the epidemiological bulletin. Unfortunately, health workers still perceive the use of the electronic tools as extra work and expect extra payment. As previous studies confirm, such attitude poses a risk to quality of care if poor performance of health personnel results from missing monetary or non-monetary incentives [32–34].

Reporting can be considered a boring and cumbersome duty for anyone working in the medical care provision sector. In our case, due to the automatic storage and uploading of all clinical information when using ALMANACH, the health worker could be partly relieved from this duty. However, in this early phase of the ALMANACH project, we were still not so confident to drop the classic paper-based reporting routine and bypass the classic flow of information to the installed HIS. Previous investigations by third research groups noticed a resistance to use electronic tools due to health worker’s fear of seeing their competence questioned [11], a phenomenon which we could not confirm in our survey. Yet, there were other external factors, which strongly influenced health worker’s attitude such as a general low work motivation, delayed salary payment and lack of supervision.

In the current study, ALMANACH was used by most of the health workers during the consultation (64.5% considering the result from the CGS). This is a promising result but not optimal and far from the results published by other groups. The IeDA project in Burkina Faso reported 95% utilization rate, possibly due to the fact that the tool has a coaching and supervision gadget embedded in their system [19], which is not the case in ALMANACH. Despite the motivation of the users remained a challenge, in the ALMANACH setting we decided to continue working without extra incentive policy. More efforts are needed and solutions sought to improve the use of the tool. Currently, the Swiss TPH is working on an enhanced version of ALMANACH, which will be more user-friendly through simplification the predetermined pathway in the decision tree without jeopardizing safety and efficacy.

2) In resource-limited countries, reports and statistics are often of poor quality and thus data exploitation for better planning and monitoring is challenging [35]. For health managers, ALMANACH exports clinical information almost real-time to the DHIS 2 HIS allowing them to promptly oversee data uploaded also from remote health facilities which are otherwise difficult to monitor. During the implementation phase in Adamawa, ALMANACH proved to be useful to investigate suspected outbreaks (e.g. measles), stock out of vitamin A and to correct the diagnosis behaviour of health workers when a suspiciously high increase of pneumonia, measles or malaria cases was reported. Moreover, in this post-conflict context, the tool was seen by the health managers as a relief to gather information from remote areas from where most of the health staff has fled.

3) We had initially many concerns regarding introducing a digital device to a population that is mostly illiterate especially about how it could change the interaction between the health worker and the patient. Our findings indicated that the use of the tablet was well accepted by the caregivers. Several mothers stated that they brought their child to the PHCC especially because ALMANACH was used; therefore, the use of the device worked as a “pulling factor” to increase the demand of health service. Our findings showed that at least in the Fulani population of Adamawa State this innovation was well accepted but we are not sure these results would be the same for other ethnic groups in other regions of Nigeria. Furthermore, implementation research is needed, such as qualitative studies assessing focal groups, to better explore this notion.

It is important to consider that ALMANACH as any CDSS, could have a beneficial effect on improving the quality of care, by promoting evidence-based medicine in rural and poor settings, is not able to unfold its full potential, if in the working environment a minimum of staff, drugs and equipment availability are not met. In our context, the health system relied on the presence of (insufficiently qualified) staff, continuously scarce drug supplies (based on the recommendations from WHO’s essential medicines list), and limited availability of basic equipment. Other major problems remain: the referral system is still very weak and most of the consultations are carried out by health staff that is not officially authorized to do it. In order to achieve noticeable impact on the main mortality indicators (e.g. Under 5 Mortality Rate) a CDSS cannot work alone without a full reinforcement of the health system.

Fever is the main reason for the consultation [36] and despite 80% of these paediatric patients were not in need of antibiotic therapy they have been treated for [23, 37]. We deem that the use of IMCI jointly with a CDSS could play a pivotal rule in the future to reduce antibiotic overprescribing, which is a major public health problem. This is particularly true for resource-limited settings where guidelines are often not followed [38], resulting in devastating effects on antibiotic resistance in these contexts [39–42]. Moreover, an adequate alternative to the Widal test should be required to avoid over-diagnosis of typhoid fever and an improper use of antibiotics.

In Adamawa State, it is foreseen that the program will be scaled up to state-wide coverage by the end of 2020. Inclusion of all 403 health facilities in the state to the program will make it possible to evaluate the global impact on the local health system as well as its cost-effectiveness. Our hypothesis is that the use of ALMANACH will save money through reduction of unnecessary prescribed medication and thus pay back the procurement cost of the tablets. Nonetheless, other important questions require answers like the full integration of our CDSS into the Adamawa’s HIS: ALMANACH tool has the potential to replace the current paper-based data collection but this process should be carried out with caution in order to avoid disastrously loss of information. Also of concern, is the currently given capacity of the Adamawa health system to take over the whole management of the project (and related costs) when the tutoring and coaching of the Swiss TPH and ICRC have come to an end.

Future investigations are planned to support the fully implementation of ALMANACH into the health system of Adamawa State and they will contribute to improve our knowledge about CDSSs and their working modality. The results from a proper implementation of IMCI in low resources settings are evident in scientific literature [4, 43, 44]. With the use of ALMANACH or other CDSSs there is the possibility to enhance the potentiality of this strategy and overwhelm the implementation problems faced in the past.

## Supporting information

S1 Text CRS Questionnaire. (DOCX)

S2 Text CGS Questionnaire. (DOCX)
