# Supplementary material for: Results from one-year use of an electronic Clinical Decision Support System in a post-conflict context: An implementation research
Source: PLoS One. 2019 Dec 2;14(12):e0225634. doi: 10.1371/journal.pone.0225634 (PMC6886837; doi:10.1371/journal.pone.0225634)
Supplement: S1 Text — (DOCX) [file pone.0225634.s001.docx]

**ALMANACH–Caretaker questionnaire**

This questionnaire aims to assess the clinical characteristics of children aged >2months to <5 years visiting clinics in which ALMANACH is used.

| ***General information*** | | | | | | | | |
| --- | --- | --- | --- | --- | --- | --- | --- | --- |
| *1.* | How old is your child?: | Year:___ Month:___ | | *2.* | | Health Facility:  🞎 Girei B 🞎 Betso  🞎 Vinikilan 🞎 Lamurde  🞎 Muva 🞎 Lokuwa | | |
|  | | | | | | | | |
| ***The visit*** | | | | | | | | |
| *3.* | Do you know what is the diagnosis of the doctor for your child? | 🞎Yes / 🞎No | | *4.* | | **If yes**, please, can you tell me the diagnosis?  **The diagnosis is:** _______________________________ | | |
| *5.* | Has the health workers explained you the health problem in a way that you understood? | 🞎 Yes  🞎 No | | *6.* | | During the visit your babe has been:  - Weighed 🞎Yes / 🞎No / 🞎 I don’t know  - Queried for the vaccination card: 🞎Yes / 🞎No 🞎 I don’t know  - Queried for deworming: 🞎Yes / 🞎No/ 🞎 I don’t know  - Queried for vitamin A: 🞎Yes / 🞎No/ 🞎 I don’t know | | |
| *7* | Was the patient vaccination status of your babe complete? | 🞎Yes / 🞎No/ 🞎 I don’t know | | *8.* | | **If no,** Was the child given the missing vaccine or asked to come back to update their vaccination status?  🞎Yes / 🞎No/ 🞎 I don’t know | | |
| *9.* | Was your child with fever? | 🞎Yes / 🞎No | | *10.* | | Was temperature taken?: 🞎Yes / 🞎No 🞎 I don’t know | | |
| *11.* | Was the child sent for a lab test? | 🞎Yes / 🞎No | | *12.* | | Which one: 🞎 Malaria 🞎 Typhoid fever  🞎 PCV 🞎 Urine exam.  🞎 HIV 🞎 stool exam.  🞎 Other: _______________________ | | |
| *13.* | Did you pay for the visit? | 🞎Yes / 🞎No | | *14.* | | Can you pay for the visit? | | 🞎Yes / 🞎No |
|  | | | | | | | | |
| ***The drugs*** | | | | | | | | |
| *15.* | Did you receive any injection in the PHCC? | 🞎Yes / 🞎No | | *16.* | | If yes, do you remember what drug?  ____________________________________  🞎 I don’t remember | | |
| *17.* | Has the doctor prescribed you drugs? | 🞎Yes / 🞎No | | *18.* | | Has someone in the health facility explained you how to use these drugs? 🞎Yes / 🞎No | | |
| *19.* | Do you know how to use these drugs? | 🞎Yes / 🞎No | |  | |  | | |
| *20.* | The drugs prescribed are:  **(ask to see the drugs or the prescription)** | *Drug Name* | | | | *Dose* | *Present in the health facility* | |
|  | Drug a |  | | | |  | 🞎Yes / 🞎No | |
|  | Drug b |  | | | |  | 🞎Yes / 🞎No | |
|  | Drug c |  | | | |  | 🞎Yes / 🞎No | |
|  | Drug d |  | | | |  | 🞎Yes / 🞎No | |
|  | Drug e |  | | | |  | 🞎Yes / 🞎No | |
|  | Drug f |  | | | |  | 🞎Yes / 🞎No | |
|  | Drug g |  | | | |  | 🞎Yes / 🞎No | |
| *21.* | Did you pay for these drugs? | 🞎Yes / 🞎No | | | *20.* | Can you pay for the drugs? | 🞎Yes / 🞎No | |
|  | | | | | | | | |
| ***ALMANACH*** | | | | | | | | |
| *22.* | **Was the health staff using a device (tablet) during the visit?** | 🞎Yes / 🞎No | | | |  | | |
| *23.* | If you reply YES at the question 21 , what is your feeling about the use of this device (*Go for the open question and then with all the other multiple choices*) |  |  |  |  |  |  |  |
|  | With the tablet the doctor is asking me much more questions  🞎True / 🞎 False | | With the tablet the doctor is examining my child in a much more careful way  🞎True / 🞎 False | | | | | |
|  | With the tablet I wait much more in the waiting room  🞎True / 🞎 False | |  | | | | | |
| *24.* | Finally, are you comfortable that the health worker use a tablet to visit your child? | 🞎Yes / 🞎No | | | | **If no**, please, could you tell us the reason? | | |
|  | | | | | | | | |
| ***Referrals*** | | | | | | | | |
| *25.* | After the consultation, did the doctor ask you to go to the hospital? | 🞎Yes / 🞎No | | *26.* | | If he asked, will you go? 🞎Yes / 🞎No  If you did not go, why? ______________ | | |
|  |  |  | |  | |  | | |
| *27.* | In the past, was your child sent to the hospital? | 🞎Yes / 🞎No | | *28.* | | Did they go? 🞎Yes / 🞎No  If you did not go, why? ______________ | | |
|  |  | | | | | | | |

**29. At the end of the day: What is the diagnosis on the register for this child: _________________**
