# Supplementary material for: Results from one-year use of an electronic Clinical Decision Support System in a post-conflict context: An implementation research
Source: PLoS One. 2019 Dec 2;14(12):e0225634. doi: 10.1371/journal.pone.0225634 (PMC6886837; doi:10.1371/journal.pone.0225634)
Supplement: S2 Text — (DOCX) [file pone.0225634.s002.docx]

**ALMANACH – Patient questionnaire**

code

Patient 🞎 Seen through ALMANACH

🞎 NOT seen through ALMANACH

This questionnaire aims to assess the clinical characteristics of children aged >2months to <5 years visiting clinics in which ALMANACH is used.

| ***General information*** | | | | | | | | | | | | |
| --- | --- | --- | --- | --- | --- | --- | --- | --- | --- | --- | --- | --- |
| 1. | Health facility? | 🞎 Girei B | | 🞎 Vinikilan | | | 2. | Consultation made by whom? | 🞎 CHO | | 🞎 CHEW | |
|  |  | 🞎 Lokuwa | | 🞎 Betso | | |  |  | 🞎 Nurse | | 🞎 Midwife | |
|  |  | 🞎 Muva | | 🞎 Lamurde | | |  | 🞎 Other: | __________________________ | | | |
|  |  |  | | | | | 3. | First visit or follow up? | 🞎 First visit  🞎 Unknown | | 🞎 Follow-up visit | |
| ***Patient information*** | | | | | | | | | | | | |
| 4. | Age (in months until 24 months, then years) | _____________months OR _____________years | | | | | 5. | Gender | 🞎 M  🞎 F | |  | |
| ***Complaint*** | | | | | | | | | | | | |
| 6. | What is the patient’s main complaint(s)? and for how many days? | Complaint  1)  2)  3)  4) | | | Days | Complaint  5)  6)  7)  8) | | | | Days | |  |
| ***Danger signs*** | | | | | | | | | | | | |
| 7. | **Did the HW assess if the child is** |  | | **Did the child has this danger sign?** | | |  | **Did the HW assess if the child is** |  | | **Did the child has this danger sign?** | |
|  | unable to drink or eat? | 🞎Yes / 🞎No | | 🞎Yes / 🞎No | | |  | lethargic or unconscious? | 🞎Yes / 🞎No | | 🞎Yes / 🞎No | |
|  | vomiting everything? | 🞎Yes / 🞎No | | 🞎Yes / 🞎No | | |  |  |  | |  | |
|  | convulsing now or recently? | 🞎Yes / 🞎No | | 🞎Yes / 🞎No | | |  |  |  | |  | |
| ***Symptoms*** | | | | | | | | | | | | |
| 8. | **Did the HW ask about** | |  | **What did the patient/caretaker say?** | | |  | **Did the HW ask about** |  | | **What did the patient/caretaker say?** | |
|  | **General symptoms?** | | |  | | |  | **Gastro-intestinal symptoms?** | | |  | |
|  | Fever | 🞎Yes / 🞎No | | 🞎Yes / 🞎No | | |  | Diarrhea | 🞎Yes / 🞎No | | 🞎Yes / 🞎No | |
|  | Fever duration | 🞎Yes / 🞎No | | ____________days | | |  | Diarrhea duration | 🞎Yes / 🞎No | | ____________days | |
|  | **Respiratory symptoms?** | | |  | | |  | Stool frequency | 🞎Yes / 🞎No | | _________(stools/ day) | |
|  | Cough | 🞎Yes / 🞎No | | 🞎Yes / 🞎No | | |  | Blood in stool | 🞎Yes / 🞎No | | 🞎Yes / 🞎No | |
|  | Cough duration | 🞎Yes / 🞎No | | ____________days | | |  | Vomiting | 🞎Yes / 🞎No | | 🞎Yes / 🞎No | |
|  | Runny nose | 🞎Yes / 🞎No | | 🞎Yes / 🞎No | | |  | Drink poorly, not able to drink | 🞎Yes / 🞎No | | 🞎Yes / 🞎No | |
|  | Conjunctivitis | 🞎Yes / 🞎No | | 🞎Yes / 🞎No | | |  | Drinks eagerly, thirsty | 🞎Yes / 🞎No | | 🞎Yes / 🞎No | |
|  | Difficult breathing | 🞎Yes / 🞎No | | 🞎Yes / 🞎No | | |  |  |  | |  | |
|  |  |  | |  | | |  |  |  | |  | |
|  | **ENT symptoms?** |  | |  | | |  | **Other symptoms?** |  | |  | |
|  | Ear pain | 🞎Yes / 🞎No | | 🞎Yes / 🞎No | | |  | Skin lesion | 🞎Yes / 🞎No | | 🞎Yes / 🞎No | |
|  | Bilateral ear pain | 🞎Yes / 🞎No | | 🞎Yes / 🞎No | | |  | Measles during the last 3 months | 🞎Yes / 🞎No | | 🞎Yes / 🞎No | |
|  | Ear pain duration | 🞎Yes / 🞎No | | __________ (days) | | |  | Dysuria |  | | 🞎Yes / 🞎No | |
|  | Ear discharge | 🞎Yes / 🞎No | | 🞎Yes / 🞎No | | |  | Other pain (specify): __________________  __________________ | 🞎Yes / 🞎No | | 🞎Yes / 🞎No | |
|  | Ear discharge duration | 🞎Yes / 🞎No | | __________ (days) | | |  |  |  | |  | |
|  | Sore throat | 🞎Yes / 🞎No | | 🞎Yes / 🞎No | | |  |  |  | |  | |
| ***Physical examination (ask if you don’t full understand what the Health worker is doing)*** | | | | | | | | | | | | |
| 9. | **Did the HW assess signs regarding:** | | | **What was the result?** | | |  | **Did the HW assess signs regarding:** | | | **What was the result?** | |
|  | **General status?** |  | |  | | |  | **Respiratory status?** |  | |  | |
|  | Pallor | 🞎Yes / 🞎No | | 🞎Yes / 🞎No | | |  | Respiratory rate | 🞎Yes / 🞎No | | ________(breaths/min) | |
|  | Temperature | 🞎Yes / 🞎No | | __________________(°C) | | |  | Chest sounds with stethoscope | 🞎Yes / 🞎No | | 🞎Normal / 🞎Abnormal Observation: __________ | |
|  | Child’s weight | 🞎Yes / 🞎No | | __________________(kg) | | |  | Chest indrawing | 🞎Yes / 🞎No | | 🞎Yes / 🞎No | |
|  | Child’s height | 🞎Yes / 🞎No | | _________________(cm) | | |  | Stridor |  | | 🞎Present / 🞎Absent | |
|  | Mid-upper arm circumference (MUAC) | 🞎Yes / 🞎No | | _________________(mm) | | |  | Cyanosis | 🞎Yes / 🞎No | | 🞎Yes / 🞎No | |
|  | Oedema |  | | 🞎Yes / 🞎No | | |  | Wheezing | 🞎Yes / 🞎No | | 🞎Yes / 🞎No | |
|  | **Hydration status?** |  | |  | | |  |  | If yes, observed through: | | | |
|  | Sunken eyes | 🞎Yes / 🞎No | | 🞎Yes / 🞎No | | |  |  | 🞎Direct listening  🞎Auscultation | | | |
|  | Restless, irritable | 🞎Yes / 🞎No | | 🞎Yes / 🞎No | | |  | **Cutaneous status?** |  | |  | |
|  | Absence of tears, dry mucus membranes | 🞎Yes / 🞎No | | 🞎Yes / 🞎No | | |  | Skin lesion | 🞎Yes / 🞎No | | 🞎Yes / 🞎No | |
|  | Skin pinch | 🞎Yes / 🞎No | | 🞎Yes / 🞎No | | |  |  |  | | If Yes, type and location of lesion: _____________________ | |
|  | **ENT status?** |  | |  | | |  | **Eyes status** |  | |  | |
|  | Mastoid percussion | 🞎Yes / 🞎No | | 🞎Painful / 🞎Not painful | | |  | Pus draining from the eyes | 🞎Yes / 🞎No | | 🞎Yes / 🞎No | |
|  | Nodes palpation |  | | Adenopathy 🞎Yes / 🞎No | | |  | Clouding of cornea | 🞎Yes / 🞎No | | 🞎Yes / 🞎No | |
|  | Throat and mouth mucosa | 🞎Yes / 🞎No | | 🞎Ulcers / 🞎Thrush / 🞎Other: ______________ | | |  | **Other sign (specify):** __________________ | 🞎Yes / 🞎No | | 🞎Yes / 🞎No | |
|  | Otoscopy | 🞎Yes / 🞎No | | Otitis (red and bulging tympanum) 🞎Yes / 🞎No | | |  |  |  | |  | |
|  | Direct observation of ear without otoscope | 🞎Yes / 🞎No | | Ear discharge 🞎Yes / 🞎No | | |  |  |  | |  | |

| ***Diagnostic tests*** | | | | | | | | | | | |
| --- | --- | --- | --- | --- | --- | --- | --- | --- | --- | --- | --- |
| 11. | **Diagnostic tests ordered:** | |  | **Test done? If done, provide result** | |  | **Diagnostic tests ordered:** | | |  | **Test done? If done, provide result** |
|  | Typhoid test | | 🞎Yes / 🞎No | 🞎Yes / 🞎No – Result:_______________ | |  | Urinary test | | | 🞎Yes / 🞎No | 🞎Yes / 🞎No – Result:_______________ |
|  | Tuberculosis test | | 🞎Yes / 🞎No | 🞎Yes / 🞎No – Result:_______________ | |  | Strep test (GAS) | | | 🞎Yes / 🞎No | 🞎Yes / 🞎No – Result:_______________ |
|  | HIV test | | 🞎Yes / 🞎No | 🞎Yes / 🞎No – Result:_______________ | |  | Stool examination | | | 🞎Yes / 🞎No | 🞎Yes / 🞎No – Result:_______________ |
|  | Malaria test | | 🞎Yes / 🞎No | 🞎Yes / 🞎No – Result:_______________ | |  | Other tests ordered (specify)?__________ | | | 🞎Yes / 🞎No | 🞎Yes / 🞎No – Result:_______________ |
| ***Other information checked*** | | | | | | | | | | | |
| 12. | **Did the HW check the following?** | | | **Was the status ok?** | |  | **Did the HW check the following?** | | | | **Was the status ok?** |
|  | Immunization status | | 🞎Yes / 🞎No | 🞎Yes / 🞎No | |  | Last deworming | | | 🞎Yes / 🞎No | 🞎Yes / 🞎No |
|  | Last vitamin A prescription | | 🞎Yes / 🞎No | 🞎Yes / 🞎No | |  | Other:_____________ | | | 🞎Yes / 🞎No | 🞎Yes / 🞎No |
| ***Diagnostic classification as per HW*** | | | | | | | | | | | |
| ***Acute diseases*** | | | | | | | | | | | |
| **13 What is the diagnosis (or diagnoses) for the HW (please ask HW and be careful in reporting):** | | | | | | | | | | | |
| ***Diagnosis 1:*** | | | | | | | | | | | |
| ***Diagnosis 2:*** | | | | | | | | | | | |
| ***Diagnosis 3:*** | | | | | | | | | | | |
| ***Diagnosis 4:*** | | | | | | | | | | | |
| ***Diagnosis 5:*** | | | | | | | | | | | |
| ***Treatment prescribed (Ask for the treatment given and the dose)*** | | | | | | | | | | | |
| **14. Antibiotics 🞎Yes / 🞎No** | | | | | | | | | | | |
|  | |  |  |  | |  |  |  | | |  |
| a) | | Name: | | Dose | | | | 🞎 tablet / 🞎 syrup 🞎 IM | | | Duration: ______ (days) |
| b) | | Name: | | Dose | | | | 🞎 tablet / 🞎 syrup 🞎 IM | | | Duration: ______ (days) |
| c) | | Name: | | Dose | | | | 🞎 tablet / 🞎 syrup 🞎 IM | | | Duration: ______ (days) |
| e) | | Name: | | Dose | | | | 🞎 tablet / 🞎 syrup 🞎 IM | | | Duration: ______ (days) |
| **15.Antimalarials 🞎Yes / 🞎No** | | | | | | | | | | | |
| a) | | Name: | | Dose | | | | 🞎 tablet / 🞎 syrup 🞎 IM | | | Duration: ______ (days) |
| b) | | Name: | | Dose | | | | 🞎 tablet / 🞎 syrup 🞎 IM | | | Duration: ______ (days) |
| c) | | Name: | | Dose | | | | 🞎 tablet / 🞎 syrup 🞎 IM | | | Duration: ______ (days) |
| **16. Pain killers/Febrifuges (including Anti-inflammatory steroid medicine) 🞎Yes / 🞎No** | | | | | | | | | | | |
| a) | | Name: | | Dose | | | | 🞎 tablet / 🞎 syrup 🞎 IM | | | Duration: ______ (days) |
| b) | | Name: | | Dose | | | | 🞎 tablet / 🞎 syrup 🞎 IM | | | Duration: ______ (days) |
| c) | | Name: | | Dose | | | | 🞎 tablet / 🞎 syrup 🞎 IM | | | Duration: ______ (days) |
| d) | | Name: | | Dose | | | | 🞎 tablet / 🞎 syrup 🞎 IM | | | Duration: ______ (days) |
| **17. Dehydration treatment plan 🞎Yes / 🞎No** | | | | | | | | | | | |
| If yes: | | 🞎 A, 🞎 B or 🞎 C? | | 🞎 Oral (ORS)/ 🞎 Naso-gastric / 🞎 IV | | | | Zinc 🞎Yes / 🞎No | | |  |
| **22. Bronchodilators 🞎Yes / 🞎No** | | | | | | | | | | | |
| a) | | Name: | | Dose | | | | 🞎 tablet / 🞎 syrup 🞎 IM | | | Duration: ______ (days) |
|  | |  | |  | | | |  | | |  |
| **18. Albendazole/Vitamin A 🞎Yes / 🞎No** | | | | | | | | | | | |
|  | | Vitamin A 🞎Yes / 🞎No | | | | | | Deworming treatment (albendazole) 🞎Yes / 🞎No | | | |
| **19. Anaemia and malnourishment treatment 🞎Yes / 🞎No** | | | | | | | | | | | |
|  | | Ready to Use Therapeutic Food 🞎Yes / 🞎No | | Duration: ______ (days) | | | | Iron 🞎Yes / 🞎No | | | Duration: ______ (days) |
|  | | Ready to Use Supplementary Food 🞎Yes / 🞎No | | Duration: ______ (days) | | | |  | | |  |
| **20. Other drugs** | | | | | | | | | | | |
|  | | Folic acid 🞎Yes / 🞎No | | Multivitamin 🞎Yes / 🞎No | | | | Tetracycline eye ointment 🞎Yes / 🞎No | | | |
| a) | | Name: | | Dose | | | | 🞎 tablet / 🞎 syrup 🞎 IM | | | Duration: ______ (days) |
| b) | | Name: | | Dose | | | | 🞎 tablet / 🞎 syrup 🞎 IM | | | Duration: ______ (days) |
| c) | | Name: | | Dose | | | | 🞎 tablet / 🞎 syrup 🞎 IM | | | Duration: ______ (days) |
| d) | | Name: | | Dose | | | | 🞎 tablet / 🞎 syrup 🞎 IM | | | Duration: ______ (days) |
| e) | | Name: | | Dose | | | | 🞎 tablet / 🞎 syrup 🞎 IM | | | Duration: ______ (days) |
| f) | | Name: | | Dose | | | | 🞎 tablet / 🞎 syrup 🞎 IM | | | Duration: ______ (days) |
| g) | | Name: | | Dose | | | | 🞎 tablet / 🞎 syrup 🞎 IM | | | Duration: ______ (days) |
| **21. Non-pharmacological treatment? (Home remedy)** | | | | | | | | | | | |
| a) | | Name: | | | | | | | | | |
| b) | | Name: | | | | | | | | | |
| c) | | Name: | | | | | | | | | |
| 22. | | **Does the child need referral?** | | 🞎Yes / 🞎No | If yes, urgently?  🞎Yes / 🞎No | | | | Pre-referral treatment: __________________________________ | | |
|  | | If the child is not referred, why?: ________________________________________________ | | | | | | | | | |
